# Female genital mutilation of a karyotypic male presenting as a female with delayed puberty

**DOI:** 10.1186/1472-6874-6-6

**Published:** 2006-03-29

**Authors:** M Ellaithi, T Nilsson, D Gisselsson, A Elagib, H Eltigani, I Fadl-Elmula

**Affiliations:** 1 Institute of Endemic Diseases, University of Khartoum, Khartoum, Sudan; 2 International University of Africa, Faculty of Medicine and Health Sciences, Khartoum, Sudan; 3 The Orchids Society for Congenitally Malformed Children. Khartoum, Sudan; 4 Department of Clinical Genetics, Lund University, Lund, Sweden; 5 Tropical Medical Research Institute, Khartoum, Sudan; 6 Department of Anatomy, Faculty of Medicine, University of Khartoum, Khartoum, Sudan; 7 Faculty of Medical Laboratory Sciences, Al Neelain University, Khartoum, Sudan; 8 Al Neelain Medical Centre, Al Neelain University, Khartoum, Sudan

## Abstract

**Background:**

Female genital mutilation (FGM) is commonly practiced mainly in a belt reaching from East to West Africa north of the equator. The practice is known across socio-economic classes and among different ethnic, religious, and cultural groups. Few studies have been appropriately designed to measure the health effects of FGM. However, the outcome of FGM on intersex individuals has never been discussed before.

**Case presentation:**

The patient first presented as a female with delayed puberty. Hormonal analysis revealed a normal serum prolactin level of 215 Mu/L, a low FSH of 0.5 Mu/L, and a low LH of 1.1 Mu/L. Type IV FGM (Pharaonic circumcision) had been performed during childhood. Chromosomal analysis showed a 46, XY karyotype and ultrasonography verified a soft tissue structure in the position of the prostate.

**Conclusion:**

FGM pose a threat to the diagnosis and management of children with abnormal genital development in the Sudan and similar societies.

## Background

Female circumcision, or female genital mutilation (FGM), is defined by the World Health Organization as "all procedures involving partial or total removal of the external female genitalia or other injury to the female genital organs, whether for cultural or other non-therapeutic reasons" [1]. Girls are commonly circumcised between the ages of 4 and 10 years, but in some communities the procedure may be performed on infants, or it may be postponed until just before marriage or even after the birth of the first child [2]. Female circumcision is practiced in 28 countries in the sub-Saharan and northeastern regions of Africa [3]. It affects over 132 million girls and women worldwide, with an additional two million girls at risk of some form of the practice each year [4]. The practice is known across socioeconomic classes and among different ethnic and cultural groups, including Christians, Muslims, Jews, and followers of indigenous African religions [5].

Based on the amount of tissue removed, Toubia has classified FGM into four main types: Type I, which is known in Sudan as "sunna", represents the excision of a part or the entire clitoris. Type II involves clitoridectomy and excision of parts of the labia minora. Types III and IV are called infibulations. Type III represents clitoridectomy, removal of the labia minora, excision of the labia majora, and stitching of the anterior two thirds of the labia majora leaving a small posterior opening. Type IV, Pharaonic circumcision or total infibulation, refers to the complete removal of the clitoris and labia minora, excision of the labia majora, and stitching of the whole raw area leaving a very small posterior opening for the passage of urine and menstrual blood [2]. According to the National Demographic and Health Survey (1989/1990) infibulation is the predominant type of FGM practiced in Sudan throughout most of the northern, north-eastern and north-western regions, with a small overall decline in the 1980s, when a shift occurred from infibulation to clitoridectomy [6]. Although the practice was originally outlawed in Sudan in 1946 and again in 1974, the United Nations has pointed out that "Sudan has the highest prevalence of female genital mutilation in the world" [7,8]. More than 90% of women in Sudan have undergone FGM. Tradition and social pressure are the main motives for performing FGM [10].

FGM is usually performed by a layperson with limited anatomical knowledge and medical training; it is an invasive and painful surgical procedure that is usually performed without anesthesia, or with local anesthesia, often resulting in serious psychological and medical complications for the young girls. Immediate complications include hypovolaemic shock, hemorrhage, infection, tetanus, and damage to the urethra or anus. Late complications include keloid formation, dermoid cyst, dyspareunia, pelvic infection, and pregnancy complications [11]. Furthermore, there is an association between the anatomical extent of FGM and primary infertility [12]. When FGM is performed on children with abnormal genitalia (e.g. ambiguous genitalia, micropenis, and hypospadia), the consequences are likely to be even more catastrophic, resulting in irreversible medical problems.

## Case presentation

### Case history

**Figure 1:**
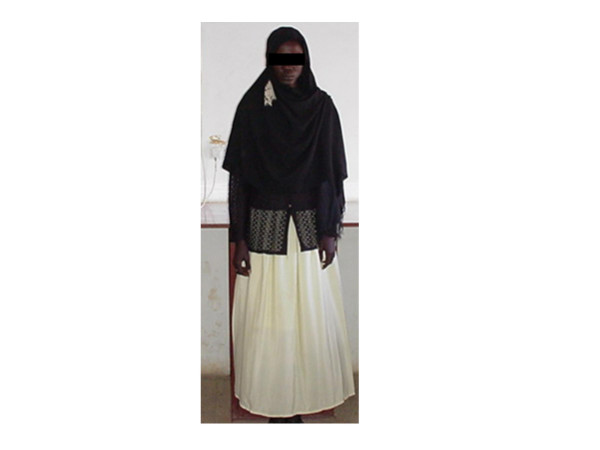
The patient dressed as a female.

**Figure 2:**
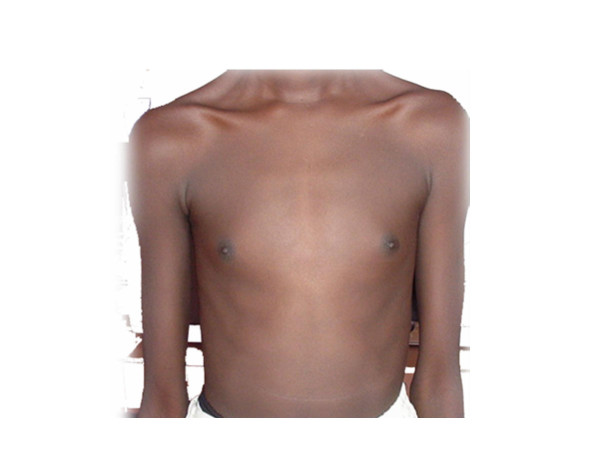
The Patient's chest: note the wide distance between breast nipples and the absence of breasts.

**Figure 3:**
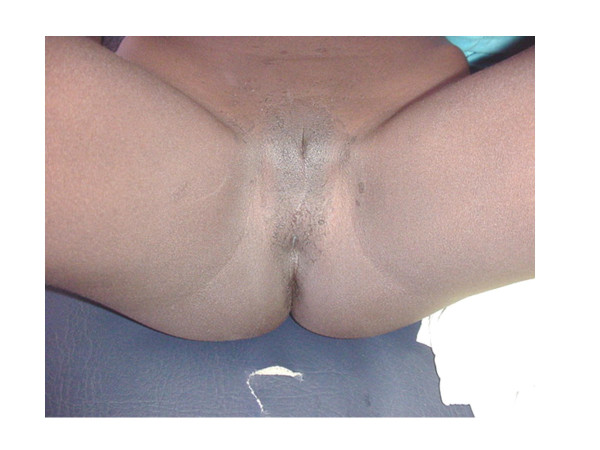
The patient's external genitalia.

A 21-year-old Sudanese patient, socially assigned as female, was referred from her gynaecologist as a case of delayed puberty for chromosomal analysis. The patient was born in one of the small towns in the centre of Sudan. A midwife decided the sex as female at the time of birth. Thus, she was given a female name and raised as a female. The patient thinks of her self as a female and was very shy to tell if she was attracted to males or females, which could be due to her cultural background. On examination she was dressed in female dress, with complete absence of facial hair (Figure 1), she was rather muscular, had broad shoulders with wide distance between the nipples, and absence of breasts or breast buds (Figure 2). The patient was about 175 cm tall and had female voice. Examination of the external genitalia showed Type IV FGM (Figure 3). There was neither pubic hair nor increased pigmentation in the genital area. Per rectum examination raised the suspicion of a prostate. Hormonal analysis revealed a serum prolactin level of 215 Mu/L, FSH of 0.5 Mu/L, and LH of 1.1 Mu/L (normal values: serum prolactin: 60–370 Mu/L, serum FSH: 1–8 Mu/L, serum LH: 2–10 Mu/L).

### Cytogenetic analysis

A peripheral blood sample of the patient was subjected to short-term culture in RPMI 1640 medium for three days, treated with Colcemide and hypotonic solution, and then fixed with methanol and acetic acid according to standard procedures. The harvested cells were dropped on clean slides and stained with Wright's stain, for chromosome banding [13]. The clonality criteria and the karyotypic descriptions were according to the ISCN (1995) recommendations [14].

**Figure 4:**
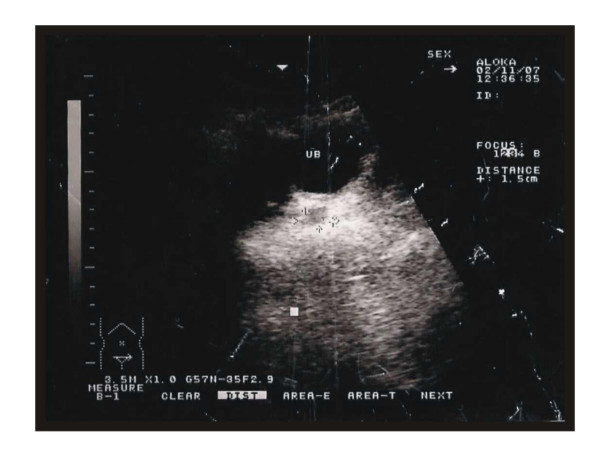
Ultra sound showing a mass in the prostate's position.

Cytogenetic analysis showed a normal male karyotype, 46, XY [27]. Ultrasound examination confirmed the presence of a 15 × 15 mm diameter soft tissue mass in the position of the male prostate (Figure 4).

## Conclusion

Although the two terms, sex and gender, are not identical, they are often used interchangeably in the language. Sex is defined based on gonads, or potential gonads, either phenotypically or genotypically. The sex of a child is usually assigned at birth based on the external genital appearance, while gender refers to the socially constructed roles and responsibilities assigned to women and men in a given culture. Thus, gender is distinct from sex, which is biologically determined. The gender of the patient described here was female. The patient's sex, on the other hand, was clearly ambiguous and the precise diagnosis remains unclear. According to her history, she had been classified as a girl at birth, but there were no records available of the configuration of her external genitalia. The patient underwent type IV FGM (Pharaonic circumcision) at an early age. This might have limited the possibility of clinical assessment, although the configuration of the external genitalia is not always essential for a correct diagnosis, as over-masculinized female pseudohermaphrodites can be similar to under-masculinized male pseudohermaphrodites. The male chromosome complement suggests that the patient suffered from a type of male pseudohermaphroditism, such as complete or incomplete androgen insensitivity syndrome. The low LH and FSH values could support this diagnosis, but the absence of breast development and the complete absence of pubic hair do not support it. The low LH and FSH could also suggest a delayed puberty condition. However it cannot be excluded that ectopic testicular tissue in or close to the labia majora was excised during the FGM process, thus explaining the absence of puberty signs. Moreover, one cannot exclude non-intersex abnormalities of the male genital organs such as grave hypospadias or conditions of hypogonadotropic hypogonadism such as Kallmann syndrome.

Cultural differences in dealing with intersexuality and other types of abnormal genitalia do not only influence the patient's own psychosexual development but may also affect medical decisions regarding sex assignment and consecutive management [15]. In this case it was difficult for the patient's physician to give her any information from the medical evaluation, other than that she is an infertile female. An unqualified birth attendance may misdiagnose the sex of a child with abnormal genital development. In the present case, the sex of rearing appeared to be correct, as the patient regarded herself as female. However, precise diagnosis and prognosis, including her potential response to hormonal therapy and subsequent fertility, remained unclear. The combination of FGM and the absence of normal female development probably subjected this patient two a twofold psychological trauma. Considering the international incidence of ambiguous genitalia cases and the high incidence of FGM in the Sudan, a significant group of patients with undiagnosed abnormal genitalia is expected among women subjected to FGM in this country. Unless religious leaders, government, and educationalists work together to clarify these matters, young individuals will continue to be genitally mutilated in the Sudan. However in a symposium organized by the Sudan Ministry of Health in conjunction with UNICEF, the health minister expressed the Sudanese government's commitment to eradicate the practice of female circumcision at all levels, and stressed that it is a prohibited act for all medical practitioners. Stiff penalties have been introduced for those who continue to perform the operations [8].

## Competing interests

The author(s) declare that they have no competing interests.

## Authors' contributions

ME has collected the sample, cultured it, and harvested metaphase chromosomes in the Institute of Endemic Diseases, University of Khartoum, Sudan; ME has also participated in the design and coordination of the study and drafted the manuscript. DG and TN performed the cytogenetic analysis, participated in the design of the study, and helped to draft the manuscript. HE has performed the ultrasonography, and helped to draft the manuscript. AE and IF participated in the design of the study, and helped to draft the manuscript.

## Pre-publication history

The pre-publication history for this paper can be accessed here:


